# Does forest extent affect salamander survival? Evidence from a long‐term demographic study of a tropical newt

**DOI:** 10.1002/ece3.3623

**Published:** 2017-11-12

**Authors:** Anthony Lau, Nancy E. Karraker, David Dudgeon

**Affiliations:** ^1^ School of Biological Sciences The University of Hong Kong Hong Kong SAR China; ^2^ Ocean Park Conservation Foundation Hong Kong Aberdeen, Hong Kong SAR China; ^3^ Department of Natural Resources Sciences University of Rhode Island Kingston RI USA

**Keywords:** Core terrestrial habitat, mark–recapture, *Paramesotriton hongkongensis*, robust design, Salamandridae

## Abstract

Forest loss has been associated with reduced survival in many vertebrates, and previous research on amphibians has mostly focused on effects at early life stages. *Paramesotriton hongkongensis* is a tropical newt that breeds in streams but spends up to 10 months per year in terrestrial habitats. Populations are threatened by habitat degradation and collection for the pet trade, but the cryptic terrestrial lifestyle of this newt has limited our understanding of its population ecology, which inhibits development of a species‐specific conservation plan. We conducted an eight‐year (2007–2014) mark–recapture study on four *P. hongkongensis* populations in Hong Kong and used these data to evaluate relationships between forest cover, body size, and rainfall on survival and to estimate population sizes. Hong Kong has been subjected to repeated historic territory‐wide deforestation, and thus, we wanted to determine whether there was a link between forest extent as a proxy of habitat quality and newt demography. Annual survival was positively associated with forest cover within core habitat of all populations and negatively related to body size. Mean annual survival (~60%) was similar to that of other stream‐dwelling amphibians, but varied among years and declined substantially in 2012–2013, perhaps due to illegal collection. Despite the link between forest extent and survival, population sizes declined at the most forested site by 40% and increased by 104% and 134% at two others. Forest protection and consequential secondary succession during recent decades in Hong Kong may have been responsible for persistence of *P. hongkongensis* populations.

## 1 INTRODUCTION

Forest loss has been associated with losses of a number of vertebrate taxa in tropical Asia (Castelletta, Sodhi, & Subaraj, 2000; Gibson et al., 2013; Laurance et al., 2012). Much of this previous work has focused on changes in species composition, and examinations of impacts to population demography are limited for the region. Many species with biphasic life cycles spend their early life stages in aquatic habitats and transition to terrestrial habitats as subadults. Core terrestrial habitat (*sensu* Semlitsch & Bodie, 2003) encompasses the upland habitat around breeding sites that many biphasic amphibians use for feeding, overwintering, and dispersal, and the importance of these habitats to maintaining viable populations has historically been underappreciated and overlooked (Semlitsch & Bodie, 2003). Proportions of intact core habitat around amphibian breeding sites are positively correlated with abundance and probability of occupancy, although the factors underlying this relationship, namely the linkage between habitat quality and survival, have yet to be clearly established (Gibbs, 1998; Homan et al. 2004; Peterman et al. 2011).

To date, long‐term demographic studies on newts (Family Salamandridae) have focused on temperate species (Griffiths, Sewell, & McCrea, 2010; Schmidt, Schaub, & Steinfartz, 2007), while research on tropical salamandrids is relatively scant (but see Fu, Karraker, & Dudgeon, 2013). Such geographic bias is not limited to newts, as studies on plants, invertebrates, and vertebrates from Southeast Asia, which is characterized by high species diversity and high threat levels, accounted for only 6% of the conservation literature published from 2011 to 2015 (i Marco et al., 2017; Sodhi, Koh, Brook, & Ng, 2004). Recent work on tropical salamandrids has largely centered on species discovery, distributions, and threats (Lau, Karraker, Martelli, & Dudgeon, 2017; Phimmachak, Stuart, & Sivongxay, 2012; Rowley et al., 2016), but confronting declining populations with conservation action requires an understanding of demography.

Terrestrial salamanders' response to deforestation/timber harvest in the United States is well documented: Relative counts of terrestrial plethodontids decreased by onefold to fivefold following clear‐cuts (Petranka et al. 1993; Sattler & Reichenbach, 1998; Knapp, Haas, Harpole, & Kirkpatrick, 2003); pond‐breeding ambystomatids responded mostly negatively to clear‐cuts and partial clear‐cuts in terms of adult survival, juvenile survival, and water loss (Semlitsch et al., 2009); monthly survival of *Plethodon shermani* from harvested forest plots was 6% lower than those from unharvested plots (Connette & Semlitsch, 2015). Terrestrial habitat quality is likely to be especially significant for salamandrids, which, like many terrestrial plethodontids, spend substantial portions of their life cycles on land (Fu et al., 2013; Gibbs, 1998) and whose populations are highly susceptible to forest degradation (Schmidt et al. 2005; Cushman, 2006; Denoël, 2012).

The Hong Kong newt (*Paramesotriton hongkongensis*) is a tropical newt confined to southern China (latitude: 22.25–23.65°N). It is classified as globally Near Threatened on the IUCN Red List (Lau & Chan, 2004) and included on Appendix II of the Convention on International Trade in Endangered Species (CITES, 2017) because of concerns over population declines associated with habitat degradation and overexploitation for the pet trade. Forests throughout Hong Kong have been cleared repeatedly since the 16th century, with the most recent episode occurring in the 1940s during the Japanese occupation (Dudgeon & Corlett, 2011). However, postoccupation protection of the countryside, and the establishment of an extensive country park system in the 1970s, has allowed the regrowth of extensive tropical secondary forests, which are characterized by a mixture of native and non‐native trees (e.g., *Lophostemon confertus*,* Machilus* spp., *Schefflera heptaphylla*; Dudgeon & Corlett, 2011).

Virtually nothing is known about effects of historic range‐wide deforestation and contemporary forest recovery on *P. hongkongensis*, but its fidelity to breeding sites and predictable seasonal migration patterns (Fu et al., 2013) indicate that populations of this newt would be amenable to monitoring using mark–recapture methods. Studies of the demography of this newt in Hong Kong thus offer a unique opportunity to access the recovery potential of populations of a tropical forest‐dependent amphibian.

Here, we present the results of a spatially replicated demographic study on a tropical salamandrid based on eight years of mark–recapture data. Our objectives were to identify factors that influence annual survival and determine population sizes of *P. hongkongensis* at these sites. Because *P. hongkongensis* spends up to 10 months per year (Fu et al., 2013) in core terrestrial habitat (i.e., riparian forest up to 113 m from breeding streams; Lau et al., 2017), and abundances of their insect prey on land tend to be positively associated with rainfall (Chan, Yu, Zhang, & Dudgeon, 2008; Yuen & Dudgeon, 2015), we hypothesized that annual survival of *P. hongkongensis* would be positively related to the extent of forest cover within its core habitat, as well as rainfall during their period spent on land each year.

## 2 MATERIALS AND METHODS

### 2.1 Study species

In Hong Kong, *P. hongkongensis* primarily occupies second‐ to fourth‐order rocky hill streams during the breeding season (September–March) and migrates to the adjacent riparian forest following breeding (Fu et al., 2013; Karsen, Lau, & Bogadek, 1986; Lau & Dudgeon, 1999). Female newts typically oviposit on trailing, submerged bank‐side vegetation, or among leaf litter and plant roots within slow‐flowing stream pools. Larvae develop in water and migrate into surrounding terrestrial habitat following metamorphosis, which takes several months (Kong & Tong, 1986). Juvenile newts spend an estimated 1–3 years on land before returning to the stream as reproductive adults, which exhibit site fidelity and visit the same breeding pool repeatedly (Fu et al., 2013; Kong & Tong, 1986).

### 2.2 Study sites and sampling methods

**Figure 1:**
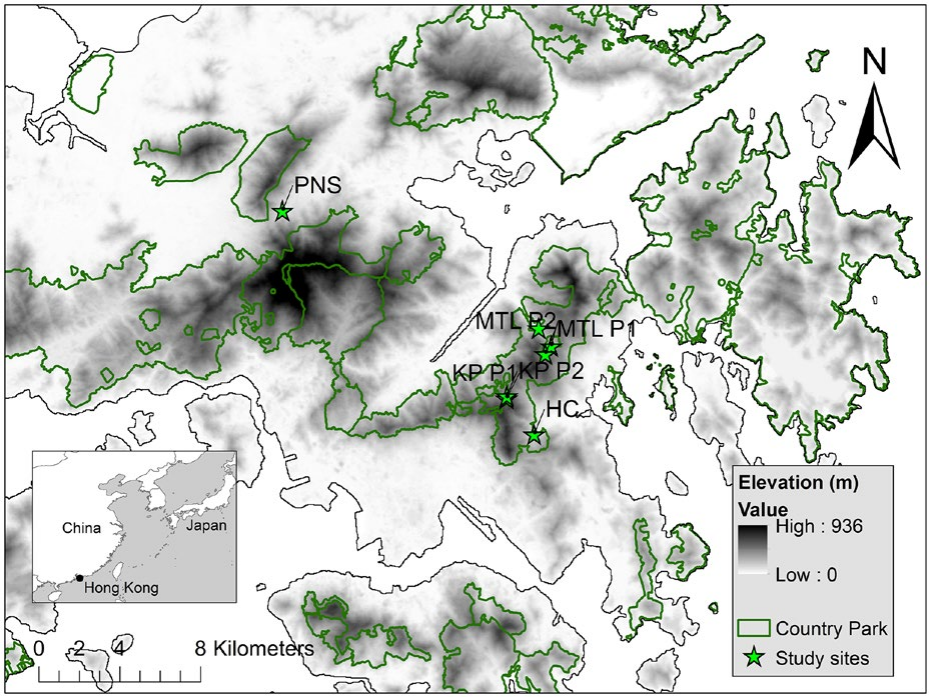
Map of Hong Kong Special Administrative Region showing the four study sites and seven breeding pools: HC, Ho Chung; KP, Kowloon Peak; MTL, Mui Tze Lam; PNS, Pak Ngau Shek

We conducted a mark–recapture study in four third‐ or fourth‐order streams that are breeding sites for *P. hongkongensis* in Hong Kong, southern China (Figure 1). Mui Tsz Lam (hereafter MTL; 129–292 m above sea level [asl]) comprised three pools along a 1.6 km stream section surrounded primarily by well‐established secondary forest; the Kowloon Peak (KP, 422 m asl) site consisted of two pools along a 20‐m stream section surrounded by a mixture of secondary forest and shrubland; Ho Chung (HC, 102 m asl) was a single stream pool surrounded by secondary forest and shrubland; and Pak Ngau Shek, (PNS, 86 m asl) was a stream pool surrounded by a mixture of abandoned agricultural land (~25%), shrubland (~25%), and secondary forest (~50%). Secondary forests in these sites are similar in age and tree composition, being less than 50 years old (except MTL, which is slightly more mature) and dominated by *Psychotria asiatica, Machilus chekiangensis,* and *Schefflera heptaphylla*. Of these four sites, only PNS is situated outside of the country park system.

**Figure 2:**
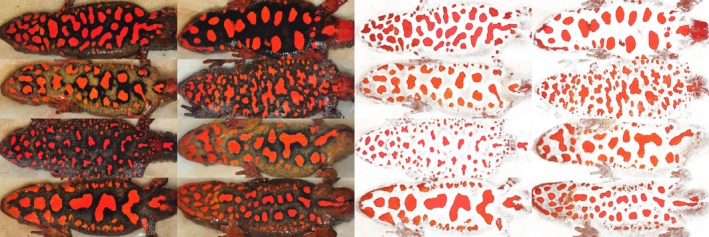
Photographs of unique ventral patterns of *Paramesotriton hongkongensis* before (left two columns) and after (right two columns) pattern extraction was carried out in Adobe Photoshop CS6

We surveyed for adult *P. hongkongensis* at seven stream pools monthly from October to March from 2007 to 2014. During each survey, two researchers searched each pool and captured all adult newts for ~3 person‐h per pool, with an additional 0.5–1 person‐h of search time during the peak breeding season (November–January, Fu et al., 2013) when newt densities were highest. Individual newts were marked (by photographing its unique ventral pattern) and measured as described in Fu et al. (2013) (Figure 2). All newts captured were breeding individuals, and those captured only during one breeding season were considered to be transients (Kendall, Nichols, & Hines, 1997; Schmidt et al., 2007).

WildID (v1.0.1) was used for computer‐assisted photograph identification to generate capture histories of individual newts (Bolger, Morrison, Vance, Lee, & Farid, 2012). Before photographs were analyzed by WildID, we extracted the ventral patterns (i.e., orange spots) of each newt from digital photographs using the “color range” function in Adobe Photoshop CS6 (Figure 2). WildID, by default, presents pictures with the 20‐highest similarity scores from the database to the user (Bolger et al., 2012). Based on our experience, the correct match occasionally does not appear among the top 20 candidates. To ensure correct identification and matching of individuals, the coding in WildID was modified by a computer programmer such that it presented the top 100 most similar individuals as potential matches.

### 2.3 Modeling survival probabilities and population size

We used the robust design (hereafter RD; Kendall et al., 1997) implemented in Program MARK ver. 8.1 (White & Burnham, 1999) to model annual apparent survival (*S*), temporary emigration (*G'* and *G”*), capture probability (*p*, set equal to recapture probability (*c*) to assume no handling effects), and derived population size (*N‐hat*). The basic structure of RD models includes multiple “closed” capture occasions (i.e., secondary occasions), between “open” survival intervals (i.e., primary occasions) (Kendall et al., 1997), which in this case correspond to sampling year (primary) and month (secondary). We did not include search effort as a parameter influencing capture rate because the effort at each site was standardized and remained constant over time (i.e., six surveys per year).

The primary parameters of interest were apparent survival, the covariates expected to affect survival (extent of forest cover; rainfall during the terrestrial phase; body length) and population size. We used a hierarchical approach to evaluate competing models to avoid unnecessary bias and imprecision in survival estimates (Lebreton, Burnham, Clobert, & Anderson, 1992; Lee et al., 2012). We started by building a fully parameterized global model with time and sex dependence in *S*,* G'*,* G”*,* p,* and *N‐hat* (Table S1). We first determined whether *p* was constant and/or varied over time (both by year and by month) and among sexes (e.g., *p*(.), *p*(sex), *p*(year)…*p*(sex + year + month). We then determined whether temporary emigration (i.e., the probability of being off the study area and unavailable for capture during a primary occasion) was “random” (*G”* = *G'*) or “Markovian” (*G'*≠*G”*) (Kendall et al., 1997) and whether it varied over time (secondary occasions within years) and among sexes. Lastly, we determined whether *S* varied by time and/or among sexes.

At all stages (i.e., before and after incorporating covariates), we identified the most parsimonious models using Akaike's information criterion, adjusted for small sample sizes (AICc/QAICs), as those with the lowest AICc/QAICc scores. Models within two AICc/QAICs units are considered to be indistinguishable from each other (Burnham & Anderson, 2002).

After determining the best model (i.e., model with the lowest ΔQAICc) for *S*,* G*”, *G',* and *p*, we denoted it as a starting model and built additional models to examine the effects of forest cover extent within the core habitat, body size, and rainfall on apparent survival. We incorporated different combinations of these factors as site, individual, or time covariates to the survival term in the starting model (e.g., *S*(forest cover + rainfall + year) vs. *S*(body size + year)) (Lee et al., 2012). Data from all four sites were pooled to estimate survival. Derived population size was estimated separately for each of the four sites. To minimize the number of parameters in our models, only simple linear relationships between apparent survival, core habitat forest cover, body length, and rainfall were considered, as more complex interactions between these factors were not expected.

### 2.4 Extent of forest cover

We hypothesized that survival of *P. hongkongensis* is influenced by the quality of terrestrial habitat around their breeding sites, and survival will be higher in sites with higher extent of forest cover. We assumed high forest cover to be a reasonable proxy for habitat quality because adult *P. hongkongensis* remained active throughout the nonbreeding season only at sites that had extensive forest cover (Lau et al., 2017). We quantified forest cover by calculating the mean satellite‐derived normalized difference vegetation index (NDVI) of the terrestrial habitat within a 113 m radius of each breeding pool. This value represents core terrestrial habitat occupied by *P. hongkongensis* during its nonbreeding season (Lau et al., 2017). NDVI is a remote sensing index used to differentiate vegetated and nonvegetated land cover types (Glenn, Huete, Nagler, & Nelson, 2008; Pettorelli et al., 2005) and is commonly used as a measure of forest cover (Carlson & Ripley, 1997; Carreiras, Pereira, & Pereira, 2006). We calculated NDVI from WorldView‐2 multispectral satellite images (resolution = 1.2 m) (courtesy of Digital Globe Foundation) using the built‐in NDVI function in ArcMAP (ESRI, version 10.1, Redlands, CA, USA). The resulting NDVI values range from −1 to 1. Positive values represent vegetation (e.g., herbaceous plants, shrubs, trees), with higher values indicating taller and denser vegetation and vice versa (Pettorelli et al., 2005). As we did not have access to multispectral satellite images from 2007 to 2014, we used images taken in January 2015 to calculate NDVI. However, because three of four sites are located within protected areas and most of the forests are >40 years postrecovery, we assumed forest cover in these sites remained relatively stable over the study duration. We verified this by comparing Google Earth images taken from 2007 to 2014 (Fig. S1). In KP, because the two stream pools are less than 10 m apart, we used the same NDVI value for both pools.

### 2.5 Rainfall during the terrestrial phase

To quantify rainfall at study sites, we obtained monthly rainfall records from the Hong Kong Observatory (http://www.hko.gov.hk). We calculated mean cumulative rainfall for the six‐month period (April–September, when ~90% of the annual rainfall in Hong Kong was accumulated) preceding a breeding season, using data from the three weather stations nearest our four study sites. For example, for the primary occasion t_2_, the 6‐month cumulative rainfall recorded between t_1_ and t_2_ was used.

### 2.6 Goodness of fit

To verify survival and recapture rate estimates obtained from RD models and to obtain breeding pool‐specific survival and recapture probability estimates, we collapsed the encounter histories to only include newt captures during a primary sampling period and ran standard open capture–recapture Cormack–Jolly–Seber (CJS) models implemented in Program MARK. As there is no standard goodness‐of‐fit test for RD models with individual covariates, we assessed model fit by running the median c‐hat procedure on the most general (full time‐dependent, *Phi*(time)*p*(time)) CJS models. For each general model, we ran the median c‐hat procedures five times with 50 intermediate points between the lower and upper bound of c‐hat. We then took the average c‐hat from the five runs and adjusted AICc values of the CJS models accordingly. Models with average c‐hat values <3 are considered to be a reasonable fit (Lebreton et al., 1992).

## 3 RESULTS

### 3.1 Factors influencing survival

Between 2007 and 2014, 42 surveys were conducted on seven primary occasions (years), with six secondary occasions (months) within each primary occasion. Surveys resulted in the capture of 9601 newts representing 3673 different individuals (1910 females, 1763 males), comprising 418 from HC, 771 from KP, 1654 from MTL, and 830 from PNS. Average (±SE) duration of stay in pools was similar among sites, ranging from 1.4 ± 0.03 month/yr (KP) to 1.9 ± 0.03 period/yr (MTL), which translates to about 42 to 57 days per visit. On average, males stayed in breeding pools longer than females (1.85 ± 0.03 versus 1.52 ± 0.02 month/yr). Newts captured from MTL and HC were longer than those captured from KP and PNS (Table 1). In total, 957 newts were recaptured (return rate = 25.6%) on two or more primary occasions, while 2,716 newts were transients. Recaptured individuals provided the data from which annual survival was modeled.

**Table 1:** Number and average body length of *Paramesotriton hongkongensis* marked and recaptured from four sites in Hong Kong (2007–2014)

Site	No. of captures	No. of recaptures	SVL (mm) ±SE
HC	418	64	69.84 ± 0.16
KP	771	148	66.08 ± 0.12
MTL	1654	538	70.06 ± 0.08
PNS	830	201	66.70 ± 0.12

HC = Ho Chung, KP = Kowloon Peak, MTL = Mui Tsz Lam, PNS = Pak Ngau Shek.

**Figure 3:**
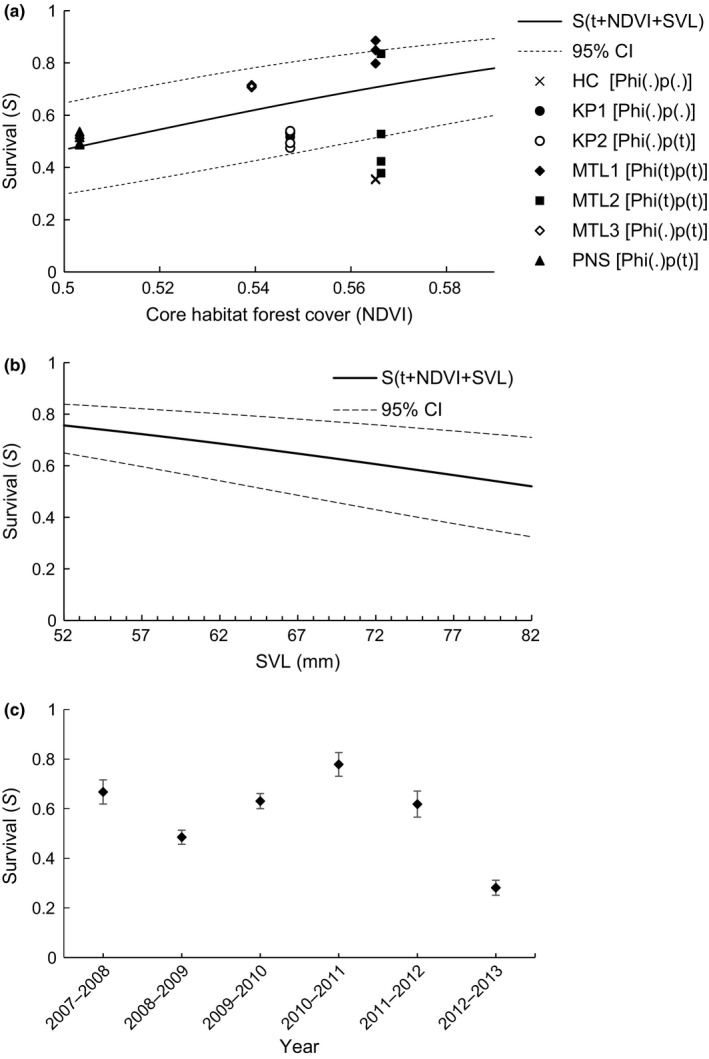
Effects of core habitat forest cover (NDVI) (a), body length (snout–vent length, SVL) (b) and year (c) on survival of *Paramesotriton hongkongensis* in Hong Kong (2007–2014) based on robust design and Cormack–Jolly–Seber (CJS) modeling. Solid and dashed lines in (a) and (b) represent predicted survival and 95% confidence interval based on beta coefficients obtained from robust design model [S(year + NDVI + SVL) G”(sex + month)  = G' p(sex + year + month)  = c f0(sex + year)] while holding all other variables constant. Symbols in (a) represent model‐averaged survival (Phi) estimates from stand‐alone CJS models from different breeding pools (best models indicated in parentheses following breeding pool codes (HC = Ho Chung, KP = Kowloon Peak, MTL = Mui Tsz Lam, PNS = Pak Ngau Shek)

**Figure 4:**
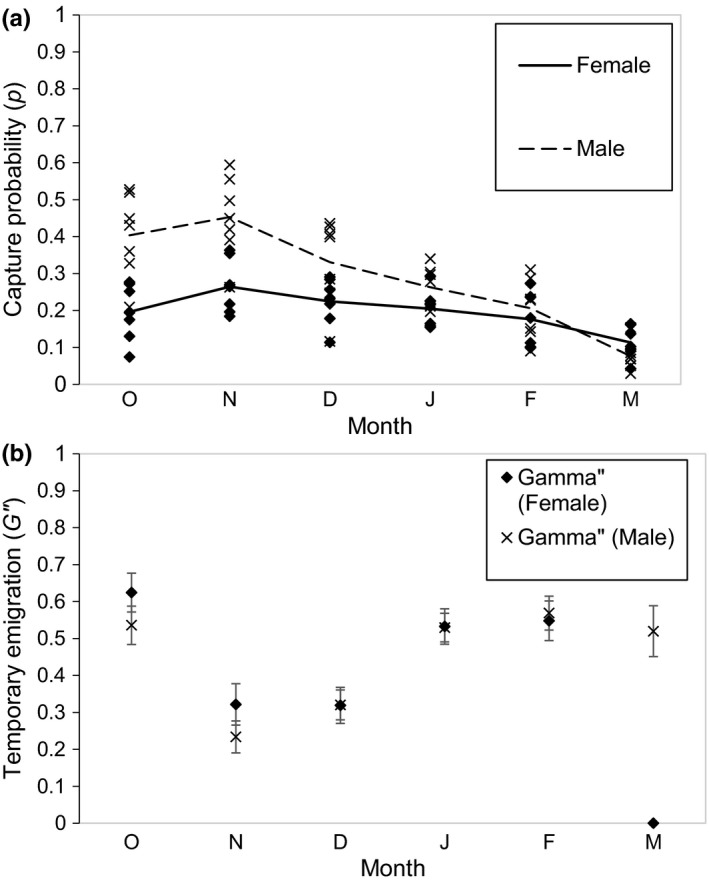
Capture probability (*p*) (a) and temporary emigration (*G”*) (b) estimates for *Paramesotriton hongkongensis* in Hong Kong (2007–2014) based on robust design mark–recapture modeling. Estimates from model [*S*(year + NDVI + SVL) *G”*(sex + month)  = *G' p*(sex + year + month)  = *c f*_*0*_(sex + year)]. Cross symbols represent males. Diamond symbols represent females. Dashed and solid lines are averages

The extent of forest cover around breeding sites and body length (SVL) was the most important factors that influenced survival in *P. hongkongensis*, as models that included NDVI and SVL consistently ranked higher than those without (Table 2). The most parsimonious RD model was [*S*(year + NDVI + SVL) *G”*(sex + month)  = *G' p*(sex + year + month)  = *c f*_*0*_(sex + year)], which indicates that apparent survival (*S*) of adult *P. hongkongensis* varied between years and was positively associated with the extent of forest cover within core habitat (β_NDVI_ = 15.46, SE = 1.46) and was negatively related to body size (SVL, β_SVL_ = −0.03, SE = 0.01). Newt *S* (survival) at the site with the highest forest cover was almost 30% higher than at the site with the least cover (Figure 3a), and *S* of the smallest newt was almost 30% higher than that of the largest individual (Figure 3b). *S* was stable across most years (average = 0.58 ± 0.04, range  = 0.28–0.77) but was considerably reduced (to >0.30) during 2012–2013 (Figure 3c). Median c‐hat procedures conducted on breeding pool‐specific CJS models showed a reasonable fit (mean c‐hat across all 7 pools = 1.38, range = 0.80–2.52). Survival and capture probability estimates from RD models were similar to those estimated from the CJS model but were more precise with smaller margins of error. Survival estimates (*Phi*) from CJS models that did not include forest cover as a covariate yielded similar survival estimates to S (Figure 3a). Capture probability (*p*) was typically <0.4 and was both time‐ (vary with sampling month and year) and sex‐dependent. Capture probability (*p*) of females was lower than that of males early in the breeding season (October–November), but from December–March, p was similar between sexes (Figure 4a). Temporary emigration was random (i.e., *G”*  = *G'*), meaning that probability of a newt being in a breeding pool during one survey was not dependent on whether it was in the breeding pool during the previous survey, but varied with month and between sexes similarly to *p* (Figure 4b).

**Table 2:** Top ten ranked robust design models (based on ΔAICc), using data from 3,674 *Paramesotriton hongkongensis* marked and recaptured from four sites in Hong Kong (2007–2014) testing the effects of core habitat forest cover (NDVI), body length (SVL), and nonaquatic period rainfall (RF) on apparent survival (S)

Model	AICc	ΔAICc	Model weight	Likelihood	Num. par.	Deviance
*S*(year + NDVI + SVL) *G”*(sex + month) = *G' p*(sex + year + month) = *c f*_*0*_(sex + year)	−9604.7	0.0	0.73	1.00	118	−9843.87
*S*(year + NDVI + SVL + RF) *G”*(sex + month) = *G' p*(sex + year + month) = *c f*_*0*_(sex + year)	−9602.6	2.1	0.26	0.36	119	−9843.87
*S*(year + NDVI) *G”*(sex + month) = *G' p*(sex + year + month) = *c f*_*0*_(sex + year)	−9594.1	10.6	0.00	0.01	117	−9831.25
*S*(year + NDVI + RF) *G”*(sex + month) = *G' p*(sex + year + month) = *c f*_*0*_(sex + year)	−9592.0	12.6	0	0	118	−9831.25
*S*(year) *G”*(sex + month) = *G' p*(sex + year + month) = *c f*_*0*_(sex + year)*	−9492.9	111.7	0	0	116	−9728.02
*S*(year + SVL) *G”*(sex + month) = *G' p*(sex + year + month) = *c f*_*0*_(sex + year)	−9492.3	112.4	0	0	117	−9729.45
*S*(year + RF) *G”*(sex + month) = *G' p*(sex + year + month) = *c f*_*0*_(sex + year)	−9490.9	113.8	0	0	117	−9728.02
*S*(year + SVL + RF) *G”*(sex + month) = *G' p*(sex + year + month) = *c f*_*0*_(sex + year)	−9490.2	114.4	0	0	118	−9729.44
*S*(year) *G”*(sex + month) *G'*(sex + month) *p*(sex + year + month) = *c f*_*0*_(sex + year)	−9487.1	117.5	0	0	126	−9742.80
*S*(sex + year) *G”*(sex + month) = *G' p*(sex + year + month) = *c f*_*0*_(sex + year)	−9485.2	119.4	0	0	122	−9732.65

AICc = Akaike's information criterion, corrected for small sample sizes; Num. par = number of parameters; *S* = survival probability; *G”*  = immigration probability; *G'* = emigration probability; *p* = capture probability; *c* = recapture probability; *f*_*0*_ = animals that were never captured.

The overall best model and starting model before the inclusion of covariates are indicated by bold font and an asterisk (*), respectively. Notations are described below and a list of all models examined is provided in the supplementary files.

Of the 957 individuals that were captured in multiple seasons, 306 (32%) were not captured in study pools during one breeding season, 79 (8%) for two seasons, and 11(1%) for at least three seasons. One female and one male from MTL were captured 32 and 26 times, respectively. Twenty individuals (10 females and 10 males, all from KP or MTL) had their first and last captures spanning our eight‐year study period. Only five of 771 individuals moved between breeding pools (all in KP, where pools were separated by less than 10 m), so we did not attempt to estimate dispersal probability.

### 3.2 Population dynamics

**Figure 5:**
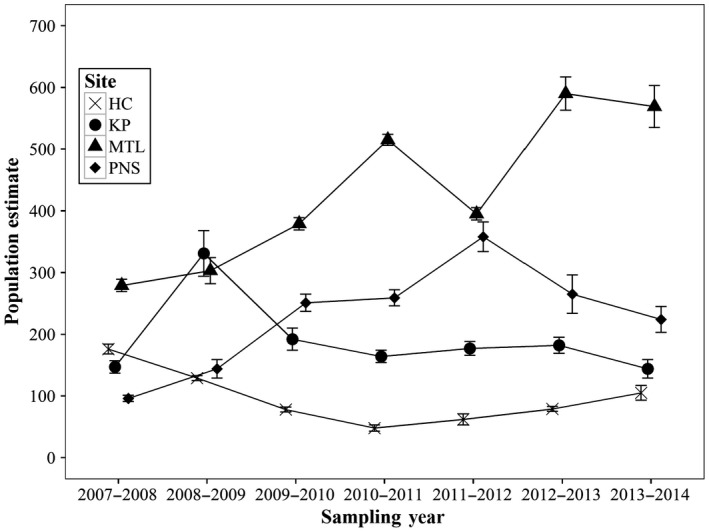
Population estimates of *Paramesotriton hongkongensis* marked and recaptured at four breeding streams in Hong Kong (2007–2014) based on robust design modeling. Error bars are 95% confidence intervals. Estimates from sites with multiple pools sampled (KP and MTL) have been combined

Based on captures over the eight‐year study period, two populations (MTL and PNS) increased in size, one remained stable, and one decreased in size (Figure 5). Population sizes at MTL and PNS increased steadily from 2007 and peaked between 2011 (+104%) and 2013 (+134%), respectively. The population in KP remained relatively stable over the study period (−2%), despite peaking at 331 newts in 2008–2009 (Figure 5). The HC population decreased by 40% over the study period, from 176 newts in 2007–2008 to 105 newts in 2013–2014 to only 48 individuals in 2010–2011. Across years, sex ratios at three sites were female‐skewed (male/female ratio: HC = 0.59; KP = 0.88; PNS = 0.81) and were male‐skewed at one site (MTL = 1.28) (Fig. S2).

## 4 DISCUSSION

Annual survival of *P. hongkongensis* increased with greater extent of forest cover around breeding pools, suggesting that this factor was a strong indicator of the quality of core terrestrial habitat. Previous studies have demonstrated impacts of forest clearance on survivorship of amphibians (Connette & Semlitsch, 2015; Otto, Roloff, & Thames, 2014; Rittenhouse, Harper, Rehard, & Semlitsch, 2008; Rothermel & Semlitsch, 2006; Todd & Rothermel, 2006) and consequential increases in risk of population extinction where forest cover has been reduced substantially (Harper, Rittenhouse, & Semlitsch, 2008), as well as life stage‐specific responses to different timber management practices (Semlitsch et al., 2009). As population dynamics in longer‐lived amphibians, such as *P. hongkongensis*, are mediated largely by the survival of adults (Berven, 1990; Gill, 1978; Wilbur, 1980), protection of undisturbed forests around breeding sites is essential for population viability. This applies particularly to species such as *P. hongkongensis* that spend, on average, over 10 months of the year away from water (Fu et al., 2013; the present study).

In contrary to our initial prediction of a positive effect of body length on survival, smaller newts had higher survival than larger individuals. Survival rates of salamander have been shown to be positively correlated with age and size (Lee et al., 2012). Unfortunately, we were not able to reliably age individual newts in the present study, as size has been proven to be a poor indicator of age for other species of newts, and skeletochronology may not be a reliable method for aging tropical species (Halliday & Verrell, 1988; Kusrini & Alford, 2006). Consistent with what was reported in an earlier study (Fu et al., 2013), newts from well‐forested sites (MTL and HC) were longer than those from less‐forested sites (KP and PNS) (Table 1). We suspect the negative effect of size on survival and lower survival observed in a well‐forested site could be related to the difference in age structure among our sites. This could also explain the population decline observed in HC.

Alternatively, or in addition, the decline of the HC population may be linked to its proximity to channelized streams. Hong Kong has an extensive network of artificial channels constructed to divert rain water from streams into reservoirs to control flooding and secure the water supply of the city's seven million occupants (Dudgeon, 1996). These channels are a source of mortality for wildlife that become entrapped as a result of their steep‐sided, smooth concrete surfaces, and are flushed downstream by high flows following heavy rain. During the breeding season, adults Hong Kong newts are often found within these artificial channels. However, these channels could be ecological traps to the newts as they contain no natural substrates and are generally shallower than natural streams where newts typically breed in. Newts trapped in these artificial channels could be more susceptible to being washed out by floods and to desiccation if the channels dry out.

The estimated survival rate of adult *P. hongkongensis* (~60%) was similar to that of *T. cristatus* in Europe (Griffiths et al., 2010) and a sympatric stream‐dwelling frog from Hong Kong (*Quasipaa spinosa*, Dicroglossidae; Chan, Shoemaker, & Karraker, 2014), but considerably lower than that of other temperate newts (*Taricha torosa*: >90% in most years, Petranka, 1998; *Salamandra salamandra*: 86%, Schmidt, Itin, & Schaub, 2014). Our apparent survival estimates may be biased low due to the high proportions of transients (>70%) in our populations (see Schmidt et al., 2007), which is not uncommon among capture–mark–recapture studies of salamanders (52% over 3 years, Schmidt et al., 2007; 49% over 5 years, Lee et al., 2012; 93% over 3 years, Unglaub, Steinfartz, Drechsler, & Schmidt, 2015). Given our relatively low capture probability (~40%), the presence of large numbers of transients may be due to some newts entering and leaving pools without being captured. Alternatively, individuals may have bred in other pools (Bucciarelli, Green, Shaffer, & Kats, 2016) or skipped breeding when environmental conditions were unfavorable (Fontenot, 1999). However, the pool‐level fidelity is remarkable in the present study in that only five of 771 individuals changed breeding pools (at a single site with two adjacent pools), suggesting that few animals moved away to breed in other pools. More frequent capture–mark–recapture surveys including multiple adjacent breeding pools would help to refine our estimates of the proportion of transients, and hence newt survival.

Although we found a positive effect of forest cover on annual survival of adult *P. hongkongensis*, we cannot causally link the increase in population size over the study period at PNS and MTL to forest recovery, as these processes operate on different time scales. The PNS population experienced the largest increase from 2007 to 2014 (Figure 5), yet its corresponding adult survival was the lowest (Figure 3a), suggesting that factors other than adult annual survival may be driving population trends. Because only adults were captured in selected breeding pools, we have no knowledge of juvenile/subadult survival rates and their influence on overall population dynamics. Subadult *P. hongkongensis* are cryptic and rarely observed in the wild (only 24% of 117 newts found during terrestrial transect surveys were subadults; Lau et al., 2017), and little is currently known about their habitat requirements or growth rates. Juvenile survival rate of salamanders is notoriously hard to estimate directly because of difficulty in relocating juveniles on an annual basis, but it is an important parameter in staged‐based demographic models built to predict population trends (Harper et al., 2008; Trenham & Shaffer, 2005). These models have shown that amphibian population growth can be sensitive to variation in terrestrial juvenile and adult survival rates (Biek et al. 2002; Vonesh & De la Cruz, 2002), and relatively small changes in adult survival can have a large impact on population growth rate (Homyack & Hass, 2009).

Although interyear fluctuation in survival was apparent (Figure 3c), the sources of this fluctuation are unclear. Survival varied with time in two out of three pools in MTL and was constant in all other pools (Figure 3a). The sudden drop in survival observed in 2012–2013 may reflect natural stochastic events, such as typhoons, that would affect all populations, or anthropogenic events, such as harvesting for the pet trade, that might impact only one or two populations. Notably, between May and June 2012, four shipments of *P. hongkongensis*, each containing 1600 individuals, were legally exported from Hong Kong to the United States (Kolby et al., 2014). Prior to the recent importation ban on Asian salamanders (USFWS, 2016), the trade in live Asian salamandrids into the United States was substantial: >770,000 individuals of >10 species imported via Hong Kong alone between 2006 and 2010, including 223,924 *P. hongkongensis* (Kolby et al., 2014). Although Hong Kong newt populations within country parks are supposedly protected by law from poaching, these parks are not patrolled at night and are easily accessible by poachers. Because of the tendency of *P. hongkongensis* to aggregate in the same breeding pools year after year, poaching could present a serious threat to the long‐term persistence of wild populations (Lau & Chan, 2004). Indeed, such vulnerability is a serious concern for other species of tropical salamandrids exhibiting similar breeding habits (e.g., *Laotriton laoensis*; Phimmachak et al., 2012). It is unclear whether the sudden decline in survival observed in 2012–2013 was related to collection during summer 2012 when newt shipments were imported to the United States, but the exported animals were unlikely to be captive bred—something that is not known to occur on any scale in Hong Kong or southern China. Although *P. hongkongensis* is, in theory, protected from poaching by the Hong Kong Wild Animals Protection Ordinance (Cap.170), charges upon violation of this regulation are brought forth in only 10–20 cases/year and mainly pertain to birds and mammals (A.S. Whitford, 2016, pers. comm.).

### 4.1 Management Implications

Approximately 23 of the 30 known breeding streams of *P. hongkongensis* occur in Hong Kong's country parks, which make up about 40% of the land area in Hong Kong and are primarily secondary forests. These populations of this forest‐dependent newt derive benefits from their occurrence in protected areas, where secondary forests continue to mature and deforestation is unlikely under current regulations. However, outside of Hong Kong, *P. hongkongensis* are known to occur only in a few isolated localities in Guangdong Province, and it is unknown whether populations persist outside of protected areas and whether enough forest remains for this species to shrive in. To further advance the conservation of this species in mainland China, we recommend 1) breeding site monitoring in selected mainland sites to access the statuses of these populations, 2) quantification of forest cover in core terrestrial habitat surrounding these breeding sites, and 3) increasing patrol in protected areas to discourage activities such as illegal logging, electrofishing, and collection of newts. Locally, we recommend establishing a buffer of at least 113 m around streams outside of protected areas where this protected species occurs (Lau et al., 2017). A similar approach should be considered for other threatened Asian salamandrids (e.g., *L. laoensis*) for which similar patterns of terrestrial habitat use and forest dependency have been observed or are suspected.

## CONFLICT OF INTEREST

None declared.

## AUTHOR CONTRIBUTION

AL collected the data. AL, NK, and DD conceived the project, analyzed the data, and wrote the article.

## ETHICAL APPROVAL

All applicable institutional and national guidelines for the care and use of animals were followed.

## References

[ece33623-bib-0001] Berven, K. A. (1990). Factors affecting population fluctuations in larval and adult stages of the wood frog (*Rana sylvatica*). Ecology, 71, 1599–1608. https://doi.org/10.2307/1938295

[ece33623-bib-0004] Biek, R. , Funk, W. C. , Maxell, B. A. , & Mills, L. S. (2002). What is missing in amphibian decline research: Insights from ecological sensitivity analysis. Conservation Biology, 16(3), 728–734.

[ece33623-bib-0002] Bolger, D. T. , Morrison, T. A. , Vance, B. , Lee, D. , & Farid, H. (2012). A computer‐assisted system for photographic mark–recapture analysis. Methods in Ecology and Evolution, 3, 813–822. https://doi.org/10.1111/j.2041-210X.2012.00212.x

[ece33623-bib-0003] Bucciarelli, G. M. , Green, D. B. , Shaffer, H. B. , & Kats, L. B. (2016). Individual fluctuations in toxin levels affect breeding site fidelity in a chemically defended amphibian. Proceedings of the Royal Society B, 283, 20160468 https://doi.org/10.1098/rspb.2016.0468 27194704 10.1098/rspb.2016.0468 PMC4892799

[ece33623-bib-0005] Carlson, T. N. , & Ripley, D. A. (1997). On the relation between NDVI, fractional vegetation cover, and leaf area index. Remote Sensing of Environment, 62, 241–252. https://doi.org/10.1016/S0034-4257(97)00104-1

[ece33623-bib-0006] Carreiras, J. M. B. , Pereira, J. M. C. , & Pereira, J. S. (2006). Estimation of tree canopy cover in evergreen oak woodlands using remote sensing. Forest Ecology and Management, 223, 45–53. https://doi.org/10.1016/j.foreco.2005.10.056

[ece33623-bib-0007] Castelletta, M. , Sodhi, N. S. , & Subaraj, R. (2000). Heavy extinctions of forest avifauna in Singapore: Lessons for biodiversity conservation in Southeast Asia. Conservation Biology, 14, 1870–1880. https://doi.org/10.1046/j.1523-1739.2000.99285.x 10.1111/j.1523-1739.2000.99285.x 35701951

[ece33623-bib-0008] Chan, H. K. , Shoemaker, K. T. , & Karraker, N. E. (2014). Demography of *Quasipaa* frogs in China reveals high vulnerability to widespread harvest pressure. Biological Conservation, 170, 3–9. https://doi.org/10.1016/j.biocon.2013.12.014

[ece33623-bib-0009] Chan, E. K. W. , Yu, Y. T. , Zhang, Y. , & Dudgeon, D. (2008). Distribution patterns of birds and insect prey in a tropical riparian forest. Biotropica, 40, 623–629. https://doi.org/10.1111/btp.2008.40.issue-5

[ece33623-bib-0010] Chanson, J. S. , Hoffman, M. , Cox, N. , & Stuart, S. N. (2008). The state of the world's amphibians In Stuart S. N., Hoffman M., Chanson J. S., Cox N. A., Berridge R. J., Ramani P. & Young B. E. (Eds.), Threatened amphibians of the world (pp. 33–44). Barcelona: Lynx Editions, IUCN and Conservation International.

[ece33623-bib-0011] CITES (Convention on International Trade in Endangered Species of Wild Fauna and Flora) (2017). Seventeenth meeting of the Conference of the Parties ‐ Proposals for amendment of Appendices I and II. Retrieved from https://cites.org/eng/cop/17/prop/index.php (accessed January 2017).

[ece33623-bib-0012] Connette, G. M. , & Semlitsch, R. D. (2015). A multistate mark–recapture approach to estimating survival of PIT‐tagged salamanders following timber harvest. Journal of Applied Ecology, 52, 1316–1324. https://doi.org/10.1111/1365-2664.12472

[ece33623-bib-0013] Crawford, J. A. , & Semlitsch, R. D. (2007). Estimation of core terrestrial habitat for stream‐breeding salamanders and delineation of riparian buffers for protection of biodiversity. Conservation Biology, 21, 152–158. https://doi.org/10.1111/cbi.2007.21.issue-1 17298521 10.1111/j.1523-1739.2006.00556.x

[ece33623-bib-0014] Cushman, S. A. (2006). Effects of habitat loss and fragmentation on amphibians: A review and prospectus. Biological Conservation, 128, 231–240. https://doi.org/10.1016/j.biocon.2005.09.031

[ece33623-bib-0015] Denoël, M. (2012). Newt decline in Western Europe: Highlights from relative distribution changes within guilds. Biodiversity and Conservation, 21, 2887–2898. https://doi.org/10.1007/s10531-012-0343-x

[ece33623-bib-0041] Di Marco, M. , Chapman, S. , Althor, G. , Kearney, S. , Besancon, C. , Butt, N. , … Watson, J.E. (2017). Changing trends and persisting biases in three decades of conservation science. Global Ecology and Conservation, 10, 32–42.https://doi.org/10.1016/j.gecco.2017.01.008

[ece33623-bib-0016] Dudgeon, D. (1996). Anthropogenic influences on Hong Kong streams. GeoJournal, 40, 53–61.

[ece33623-bib-0017] Dudgeon, D. , & Corlett, R. T. (2011). The ecology and biodiversity of Hong Kong. Hong Kong: Agriculture, Fisheries and Conservation Department, Government of Hong Kong SAR. Cosmos Books Ltd.

[ece33623-bib-0018] Fontenot, C. L. (1999). Reproductive biology of the aquatic salamander *Amphiuma tridactylum* in Louisiana. Journal of Herpetology, 33, 100–105. https://doi.org/10.2307/1565548

[ece33623-bib-0019] Fu, W. K. (2010). Population dynamics, diet and morphological variation of the Hong Kong newt (Paramesotriton hongkongensis). MPhil thesis. The University of Hong Kong, Hong Kong.

[ece33623-bib-0020] Fu, V. W. K. , Karraker, N. E. , & Dudgeon, D. (2013). Breeding dynamics, diet, and body condition of the Hong Kong Newt (*Paramesotriton hongkongensis*). Herpetological Monographs, 27, 1–22. https://doi.org/10.1655/HERPMONOGRAPHS-D-11-00010

[ece33623-bib-0021] Gibbs, J. P. (1998). Distribution of woodland amphibians along a forest fragmentation gradient. Landscape Ecology, 13, 263–268. https://doi.org/10.1023/A:1008056424692

[ece33623-bib-0022] Gibson, L. , Lynam, A. J. , Bradshaw, C. J. , He, F. , Bickford, D. P. , Woodruff, D. S. , … Laurance, W. F. (2013). Near‐complete extinction of native small mammal fauna 25 years after forest fragmentation. Science, 341, 1508–1510. https://doi.org/10.1126/science.1240495 24072921 10.1126/science.1240495

[ece33623-bib-0023] Gill, D. E. (1978). The metapopulation ecology of the red‐spotted newt, *Notophthalmus viridescens* (Rafinesque). Ecological Monographs, 48, 145–166. https://doi.org/10.2307/2937297

[ece33623-bib-0024] Glenn, E. P. , Huete, A. R. , Nagler, P. L. , & Nelson, S. G. (2008). Relationship between remotely‐sensed vegetation indices, canopy attributes and plant physiological processes: What vegetation indices can and cannot tell us about the landscape. Sensors, 8, 2136–2160. https://doi.org/10.3390/s8042136 27879814 10.3390/s8042136 PMC3673410

[ece33623-bib-0025] Griffiths, R. A. , Sewell, D. , & McCrea, R. S. (2010). Dynamics of a declining amphibian metapopulation: Survival, dispersal and the impact of climate. Biological Conservation, 143, 485–491. https://doi.org/10.1016/j.biocon.2009.11.017

[ece33623-bib-0026] Halliday, T. R. , & Verrell, P. A. (1988). Body size and age in amphibians and reptiles. Journal of Herpetology, 22, 253–265. https://doi.org/10.2307/1564148

[ece33623-bib-0027] Harper, E. B. , Rittenhouse, T. A. , & Semlitsch, R. D. (2008). Demographic consequences of terrestrial habitat loss for pool‐breeding amphibians: Predicting extinction risks associated with inadequate size of buffer zones. Conservation Biology, 22, 1205–1215. https://doi.org/10.1111/cbi.2008.22.issue-5 18717698 10.1111/j.1523-1739.2008.01015.x

[ece33623-bib-0101] Homan, R. N. , Windmiller, B. S. , & Reed, J. M. (2004). Critical thresholds associated with habitat loss for two vernal pool‐breeding amphibians. Ecological Applications, 14(5), 1547–1553.

[ece33623-bib-0028] Homyack, J. A. , & Hass, C. A. (2009). Long‐term effects of experimental forest harvesting on abundance and reproductive demography of terrestrial salamanders. Biological Conservation, 142, 110–121. https://doi.org/10.1016/j.biocon.2008.10.003

[ece33623-bib-0029] Karsen, S. J. , Lau, M. W. N. , & Bogadek, A. (1986). Hong Kong amphibians and reptiles. British Hong Kong: Urban Council.

[ece33623-bib-0030] Kendall, W. L. , Nichols, J. D. , & Hines, J. E. (1997). Estimating temporary emigration using capture‐recapture data with Pollock's robust design. Ecology, 78, 563–578.

[ece33623-bib-0031] Knapp, S. M. , Haas, C. A. , Harpole, D. N. , & Kirkpatrick, R. L. (2003). Initial effects of clearcutting and alternative silvicultural practices on terrestrial salamander abundance. Conservation Biology, 17, 752–762. https://doi.org/10.1046/j.1523-1739.2003.02061.x

[ece33623-bib-0032] Kolby, J. E. , Smith, K. M. , Berger, L. , Karesh, W. B. , Preston, A. , Pessier, A. P. , & Skerratt, L. F. (2014). First evidence of amphibian chytrid fungus (*Batrachochytrium dendrobatidis*) and Ranavirus in Hong Kong amphibian trade. PLoS ONE, 9(3), e90750 https://doi.org/10.1371/journal.pone.0090750 24599268 10.1371/journal.pone.0090750 PMC3944218

[ece33623-bib-0033] Kong, Y.‐C. , & Tong, T.‐M. (1986). The developmental stages of *Paramesotriton hongkongensis* (Myers & Leviton). Acta Herpetologica Sinica, 5, 106–118. [In Chinese].

[ece33623-bib-0034] Kusrini, M. D. , & Alford, R. A. (2006). The application of skeletochronology to estimate ages of three species of frogs in West Java. Herpetological Review, 37, 423–425.

[ece33623-bib-0035] Lau, M. W. N. , & Chan, B. P. L. (2004). Paramesotriton hongkongensis. The IUCN Red List of Threatened Species 2004: e.T59460A11945539. Gland, Switzerland. Retrieved from http://www.iucnredlist.org/details/59460/0/(Accessed July 2016).

[ece33623-bib-0036] Lau, M. W. N. , & Dudgeon, D. (1999). Composition and distribution of Hong Kong amphibian fauna. Memoirs of the Hong Kong Natural History Society, 22, 1–79.

[ece33623-bib-0037] Lau, A. , Karraker, N. E. , Martelli, P. , & Dudgeon, D. (2017). Delineation of core terrestrial habitat for conservation of a tropical salamander: The Hong Kong newt (*Paramesotriton hongkongensis*). Biological Conservation, 209, 76–82. https://doi.org/10.1016/j.biocon.2017.02.017

[ece33623-bib-0038] Laurance, W. F. , Useche, D. C. , Rendeiro, J. , Kalka, M. , Bradshaw, C. J. , Sloan, S. P. , … Alvarez, P. (2012). Averting biodiversity collapse in tropical forest protected areas. Nature, 489, 290–294. https://doi.org/10.1038/nature11318 22832582 10.1038/nature11318

[ece33623-bib-0039] Lebreton, J.‐D. , Burnham, K. P. , Clobert, J. , & Anderson, D. R. (1992). Modeling survival and testing biological hypotheses using marked animals. A unified approach with case studies. Ecological Monographs, 62, 67–118. https://doi.org/10.2307/2937171

[ece33623-bib-0040] Lee, D. E. , Bettaso, J. B. , Bond, M. L. , Bradley, B. R. , Tietz, J. R. , & Warzybok, P. M. (2012). Growth, age at maturity, and age‐specific survival of the Arboreal salamander (*Aneides lugubris*) on Southeast Farallon Island, California. Journal of Herpetology, 46, 64–71. https://doi.org/10.1670/10-282

[ece33623-bib-0042] Otto, C. R. V. , Roloff, G. J. , & Thames, R. E. (2014). Comparing population patterns to processes: Abundance and survival of a forest salamander following habitat degradation. PLoS ONE, 9, e93859 https://doi.org/10.1371/journal.pone.0093859 24718498 10.1371/journal.pone.0093859 PMC3981728

[ece33623-bib-0102] Peterman, W. E. , Crawford, J. A. , & Semlitsch, R. D. (2011). Effects of even‐aged timber harvest on stream salamanders: Support for the evacuation hypothesis. Forest Ecology and Management, 262(12), 2344–2353.

[ece33623-bib-0043] Petranka, J. W. (1998). Salamanders of the United States and Canada. Washington DC, and London: Smithsonian Institution Press.

[ece33623-bib-0103] Petranka, J. W. , Eldridge, M. E. , & Haley, K. E. (1993). Effects of timber harvesting on southern Appalachian salamanders. Conservation biology, 7(2), 363–370.

[ece33623-bib-0044] Pettorelli, N. , Vik, J. O. , Mysterud, A. , Gaillard, J. M. , Tucker, C. J. , & Stenseth, N. C. (2005). Using the satellite‐derived NDVI to assess ecological responses to environmental change. Trends in Ecology and Evolution, 20, 503–510. https://doi.org/10.1016/j.tree.2005.05.011 16701427 10.1016/j.tree.2005.05.011

[ece33623-bib-0045] Phimmachak, S. , Stuart, B. L. , & Sivongxay, N. (2012). Distribution, natural history, and conservation of the Lao newt (*Laotriton laoensis*) (Caudata: Salamandridae). Journal of Herpetology, 46, 120–128. https://doi.org/10.1670/11-044

[ece33623-bib-0046] Pilliod, D. S. , Muths, E. , Scherer, R. D. , Bartelt, P. E. , Corn, P. S. , Hossack, B. R. , … Gaughan, C. (2009). Effects of amphibian chytrid fungus on individual survival probability in wild boreal toads. Conservation Biology, 24, 1259–1267. 10.1111/j.1523-1739.2010.01506.x 20412086

[ece33623-bib-0047] Rittenhouse, T. A. G. , Harper, E. B. , Rehard, L. R. , & Semlitsch, R. D. (2008). The role of microhabitats in a desiccation and survival of anurans in recently harvested oak‐hickory forest. Copeia, 2008, 807–814. https://doi.org/10.1643/CH-07-176

[ece33623-bib-0048] Rittenhouse, T. A. G. , & Semlitsch, R. D. (2007). Distribution of amphibians in terrestrial habitat surrounding wetlands. Wetlands, 27, 153–161. https://doi.org/10.1672/0277-5212(2007)27[153:DOAITH]2.0.CO;2

[ece33623-bib-0049] Rothermel, B. B. , & Semlitsch, R. D. (2006). Consequences of forest fragmentation for juvenile survival in spotted (*Ambystoma maculatum*) and marbled (*Ambystoma opacum*) salamanders. Canadian Journal of Zoology, 84, 797–807. https://doi.org/10.1139/z06-056

[ece33623-bib-0050] Rowley, J. J. L. , Chan, S. K. F. , Tang, W. S. , Speare, R. , Skerratt, L. F. , Alford, R. A. , … Campbell, R. (2007). Survey for the amphibian chytrid *Batrachochytrium dendrobatidis* in Hong Kong in native amphibians and in the international amphibian trade. Diseases of Aquatic Organisms, 78, 87–95. https://doi.org/10.3354/dao01861 18286805 10.3354/dao01861

[ece33623-bib-0051] Rowley, J. J. L. , Shepherd, C. R. , Stuart, B. L. , Nguyen, T. Q. , Hoang, H. D. , Cutajar, T. P. , … Phimmachak, S. (2016). Estimating the global trade in Southeast Asian newts. Biological Conservation, 199, 96–100. https://doi.org/10.1016/j.biocon.2016.05.001

[ece33623-bib-0052] Sattler, P. , & Reichenbach, N. (1998). The effects of timbering on *Plethodon hubrichti*: Short‐term effects. Journal of Herpetology, 32, 399–404. https://doi.org/10.2307/1565454

[ece33623-bib-0053] Schmidt, B. R. , Itin, E. , & Schaub, M. (2014). Seasonal and annual survival of the salamander *Salamandra salamandra salamandra* . Journal of Herpetology, 48, 20–23. https://doi.org/10.1670/12-056

[ece33623-bib-0104] Schmidt, B. R. , Feldmann, R. , & Schaub, M. (2005). Demographic processes underlying population growth and decline in Salamandra salamandra. Conservation biology, 19(4), 1149–1156.

[ece33623-bib-0054] Schmidt, B. R. , Schaub, M. , & Steinfartz, S. (2007). Apparent survival of the salamander *Salamandra salamandra* is low because of high migratory activity. Frontiers in Zoology, 4, 19 https://doi.org/10.1186/1742-9994-4-19 17803829 10.1186/1742-9994-4-19 PMC2020470

[ece33623-bib-0055] Semlitsch, R. D. , & Bodie, J. R. (2003). Biological criteria for buffer zones around wetlands and riparian habitats for amphibians and reptiles. Conservation Biology, 17, 1219–1228. https://doi.org/10.1046/j.1523-1739.2003.02177.x

[ece33623-bib-0056] Semlitsch, R.D. , Todd, B.D. , Blomquist, S.M. , Calhoun, A.J. , Gibbons, J.W. , Gibbs, J.P. , … Patrick, D.A (2009). Effects of timber harvest on amphibian populations: Understanding mechanisms from forest experiments. BioScience, 59, 853–862. https://doi.org/10.1525/bio.2009.59.10.7

[ece33623-bib-0057] Sodhi, N. S. , Koh, L. P. , Brook, B. W. , & Ng, P. K. L. (2004). Southeast Asian biodiversity: An impending disaster. Trends in Ecology and Evolution, 19, 654–660. https://doi.org/10.1016/j.tree.2004.09.006 16701328 10.1016/j.tree.2004.09.006

[ece33623-bib-0058] Todd, B. D. , & Rothermel, B. B. (2006). Assessing quality of clearcut habitats for amphibians: Effects on abundances versus vital rates in the southern toad (*Bufo terrestris*). Biological Conservation, 133, 178–185. https://doi.org/10.1016/j.biocon.2006.06.003

[ece33623-bib-0059] Trenham, P. C. , & Shaffer, H. B. (2005). Amphibian upland habitat use and its consequences for population viability. Ecological Applications, 15, 1158–1168. https://doi.org/10.1890/04-1150

[ece33623-bib-0060] Unglaub, B. , Steinfartz, S. , Drechsler, A. , & Schmidt, B. R. (2015). Linking habitat suitability to demography in a pond‐breeding amphibian. Frontiers in Zoology, 12, 9 https://doi.org/10.1186/s12983-015-0103-3 25977702 10.1186/s12983-015-0103-3 PMC4430901

[ece33623-bib-0061] USFWS (U.S. Fish and Wildlife Service) (2016) News Release: Service Lists 201 Salamander Species as Injurious to Help Keep Lethal Fungus Out of U.S. Retrieved from https://www.fws.gov/injuriouswildlife/pdf_files/Bsal_News_Release_F.pdf (accessed October 2016).

[ece33623-bib-0062] Vonesh, J. R. , & De la Cruz, O. (2002). Complex life cycles and density dependence: Assessing the contribution of egg mortality to amphibian declines. Oecologia, 133, 325–333. https://doi.org/10.1007/s00442-002-1039-9 28466219 10.1007/s00442-002-1039-9

[ece33623-bib-0063] White, G. C. , & Burnham, K. P. (1999). Program MARK: Survival estimation from populations of marked animals. Bird Study, 46(supplement 1), S120–S139. https://doi.org/10.1080/00063659909477239

[ece33623-bib-0064] Wilbur, H. M. (1980). Complex life cycles. Annual Review of Ecology and Systematics, 11, 67–93. https://doi.org/10.1146/annurev.es.11.110180.000435

[ece33623-bib-0065] Yuen, E. Y. L. , & Dudgeon, D. (2015). The magnitude and seasonality of aquatic insect subsidies to tropical stream riparia in Hong Kong. Aquatic Sciences, 78, 655–666.

