# Supplementary material for: Does forest extent affect salamander survival? Evidence from a long‐term demographic study of a tropical newt
Source: Ecol Evol. 2017 Nov 12;7(24):10963–73. doi: 10.1002/ece3.3623 (PMC5743689; doi:10.1002/ece3.3623)
Supplement: Supplementary file 1 [file ECE3-7-10963-s001.docx]

**Table S1.** A list of robust design models (rank based on ΔAICc), using data from 3674 *Paramesotriton hongkongensis* marked and recaptured from four sites in Hong Kong, 2007-2014, testing for the effects of core habitat forest cover (NDVI), body length (SVL), and non-aquatic period rainfall (RF) on apparent survival (S). The overall best model and starting model before the inclusion of covariates are indicated by bold font and an asterisk (*). Global model is indicated by $. Notations are described below.

| Model | AICc | ΔAICc | AICc Weights | Model Likelihood | Num. Par | Deviance |
| --- | --- | --- | --- | --- | --- | --- |
| *S*(year+NDVI+SVL) *G"*(sex+month)=*G'* *p*(sex+year+month)=*c* *f_0_*(sex+year) | -9604.7 | 0.0 | 0.73 | 1.00 | 118 | -9843.87 |
| *S*(year+NDVI+SVL+RF) *G"*(sex+month)=*G'* *p*(sex+year+month)=*c* *f_0_*(sex+year) | -9602.6 | 2.1 | 0.26 | 0.36 | 119 | -9843.87 |
| *S*(year+NDVI) *G"*(sex+month)=*G'* *p*(sex+year+month)=*c* *f_0_*(sex+year) | -9594.1 | 10.6 | 0.00 | 0.01 | 117 | -9831.25 |
| *S*(year+NDVI+RF) *G"*(sex+month)=*G'* *p*(sex+year+month)=*c* *f_0_*(sex+year) | -9592.0 | 12.6 | 0 | 0 | 118 | -9831.25 |
| *S*(year) *G"*(sex+month)=*G'* *p*(sex+year+month)=*c* *f_0_*(sex+year)$ | -9492.9 | 111.7 | 0 | 0 | 116 | -9728.02 |
| *S*(year+SVL) *G"*(sex+month)=*G'* *p*(sex+year+month)=*c* *f_0_*(sex+year) | -9492.3 | 112.4 | 0 | 0 | 117 | -9729.45 |
| *S*(year+RF) *G"*(sex+month)=*G'* *p*(sex+year+month)=*c* *f_0_*(sex+year)* | -9490.9 | 113.8 | 0 | 0 | 117 | -9728.02 |
| *S*(year+SVL+RF) *G"*(sex+month)=*G'* *p*(sex+year+month)=*c* *f_0_*(sex+year) | -9490.2 | 114.4 | 0 | 0 | 118 | -9729.44 |
| *S*(year) *G"*(sex+month) *G'*(sex+month) *p*(sex+year+month)=*c* *f_0_*(sex+year) | -9487.1 | 117.5 | 0 | 0 | 126 | -9742.8 |
| *S*(sex+year) *G"*(sex+month)=*G'* *p*(sex+year+month)=*c* *f_0_*(sex+year) | -9485.2 | 119.4 | 0 | 0 | 122 | -9732.65 |
| *S*(year) *G"*(year) *G'*(year) *p*(sex+year+month)=*c* *f_0_*(sex+year) | -9483.6 | 121.1 | 0 | 0 | 115 | -9716.61 |
| *S*(sex+year) *G"*(sex+year) *G'*(sex+year) *p*(sex+year+month)=*c* *f_0_*(sex+year) | -9482.1 | 122.6 | 0 | 0 | 132 | -9750.07 |
| *S*(year) *G"*(year)=*G'* *p*(sex+year+month)=*c* *f_0_*(sex+year) | -9480.1 | 124.6 | 0 | 0 | 110 | -9702.84 |
| *S*(sex+year) *G"*(month)=*G'* *p*(sex+year+month)=*c* *f_0_*(sex+year) | -9469.3 | 135.4 | 0 | 0 | 116 | -9704.41 |
| *S*(sex) *G"*(sex+month)=*G'* *p*(sex+year+month)=*c* *f_0_*(sex+year) | -9465.9 | 138.7 | 0 | 0 | 112 | -9692.81 |
| *S*(.) *G"*(sex+month)=*G'* *p*(sex+year+month)=*c* *f_0_*(sex+year) | -9465.4 | 139.3 | 0 | 0 | 111 | -9690.21 |
| *S*(sex+year) *G"*(.)=*G'* *p*(sex+year+month)=*c* *f_0_*(sex+year) | -9439.2 | 165.5 | 0 | 0 | 111 | -9664.00 |
| *S*(sex+year) *G"*(sex)=*G'* *p*(sex+year+month)=*c* *f_0_*(sex+year) | -9437.6 | 167.1 | 0 | 0 | 112 | -9664.46 |
| *S*(sex+year) *G"*(sex+month) *G'*(sex+month) *p*(year+month)=*c* *f_0_*(sex+year) | -8992.1 | 612.5 | 0 | 0 | 90 | -9174.00 |
| *S*(sex+year) *G"*(sex+month) *G'*(sex+month) *p*(sex+month)=*c* *f_0_*(sex+year) | -8690.2 | 914.5 | 0 | 0 | 60 | -8811.04 |
| *S*(sex+year) *G"*(year+month) *G'*(year+month) *p*(month)=*c* *f_0_*(sex+year) | -8211.2 | 1393.5 | 0 | 0 | 54 | -8319.88 |
| *S*(sex+year) *G"*(sex+month) *G'*(sex+month) *p*(sex+year)=*c* *f_0_*(sex+year) | -7176.9 | 2427.8 | 0 | 0 | 62 | -7301.79 |
| *S*(sex+year) *G"*(sex+month) *G'*(sex+month) *p*(year)=*c* *f_0_*(sex+year) | -6954.8 | 2649.9 | 0 | 0 | 55 | -7065.49 |
| *S*(sex+year) *G"*(sex+month) *G'*(sex+month) *p*(sex)=*c* *f_0_*(sex+year) | -6923.7 | 2681.0 | 0 | 0 | 50 | -7024.29 |
| *S*(sex+year) *G"*(sex+month) *G'*(sex+month) *p*(.)=*c* *f_0_*(sex+year) | -6699.7 | 2905.0 | 0 | 0 | 49 | -6798.22 |

AICc = Akaike’s Information Criterion, corrected for small sample sizes; Num. par = number of parameters; *S* = survival probability; *G”* = immigration probability; *G’* = emigration probability; *p* = capture probability; *c* = recapture probability; *f_0_* = animals that were never captured.


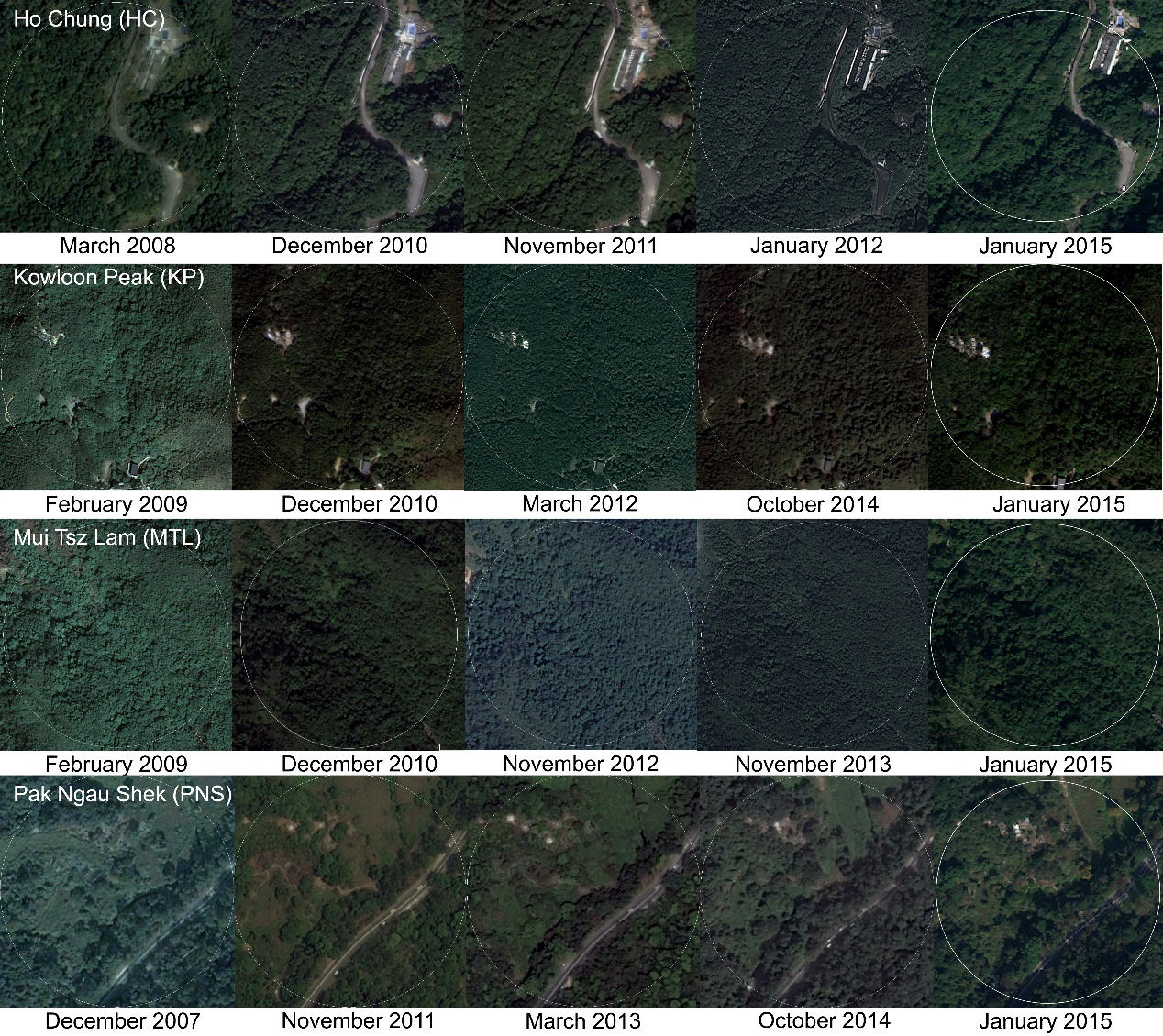


**Fig. S1.** Satellite images of core terrestrial habitat (Lau et al. 2017) surrounding four *Paramesotriton hongkongensis* breeding pools in Hong Kong, 2007-2015. The circled area is 113 m radius centered on a *P. hongkongensis* breeding pool. Images on the far right are provided by the DigitalGlobe Foundation via an imagery grant. All other images were downloaded from Google Earth Pro.

**Fig. S2.** Sex ratios of 3674 *Paramesotriton hongkongensis* captured from four breeding streams in Hong Kong (2007-2014).
